# Comparative surgical outcomes of pterional, supraorbital, and endoscopic endonasal approaches for tuberculum sellae meningiomas: a systematic review and network meta-analysis

**DOI:** 10.1186/s12893-026-03882-4

**Published:** 2026-07-02

**Authors:** Ali Msheik, Amr El Mohamad, Ali Sulaiman, Amro Alhajali, Ali Ayyad

**Affiliations:** 1 Neurosurgery, Neuroscience Institute, Hamad Medical Corporation, Doha, Qatar; 2 Department of Neurosurgery, Jordan University Hospital, Amman, Jordan; 3 Faculty of Medicine, University of Jordan, Amman, Jordan

**Keywords:** Tuberculum sellae meningioma, Pterional approach, Supraorbital keyhole, Endoscopic endonasal approach, Systematic review, Network meta-analysis

## Abstract

**Background:**

The optimal surgical corridor for tuberculum sellae meningiomas (TSMs) remains a subject of ongoing debate. While the pterional (PT) approach is the traditional gold standard, the endoscopic endonasal approach (EEA) and supraorbital (SO) keyhole approach have emerged as viable alternatives.

**Objective:**

To comparatively evaluate the surgical outcomes and complication profiles of the PT, SO, and EEA techniques for the management of TSMs.

**Methods:**

A systematic literature search was conducted across PubMed, Embase, Scopus, Web of Science, and Cochrane databases through March 2026. Frequentist random-effects network meta-analysis was performed to compare gross total resection (GTR), visual improvement, and complications. Treatment rankings were estimated using Surface Under the Cumulative Ranking Curve (SUCRA) scores.

**Results:**

Six retrospective comparative studies involving 205 patients were included. Network meta-analysis showed no statistically significant differences between approaches for gross total resection (PT vs. EEA: RR 1.21, 95% CI 0.85–1.73; SO vs. EEA: RR 1.04, 95% CI 0.86–1.24). SUCRA rankings suggested only exploratory numerical patterns, with PT ranking highest for GTR and SO showing a non-significant trend for visual improvement compared with EEA (RR 1.30, 95% CI 0.95–1.76). These rankings were not supported by statistically significant pairwise differences and should be interpreted cautiously because of sparse evidence, retrospective study designs, and wide confidence or prediction intervals. Secondary outcomes, including cerebrospinal fluid leak, diabetes insipidus, and tumor recurrence, were imprecisely estimated and broadly comparable across approaches.

**Conclusion:**

Available retrospective comparative evidence suggests that pterional, supraorbital, and endoscopic endonasal approaches may provide broadly comparable outcomes for TSMs, but the certainty of evidence is limited. No approach demonstrated statistically significant superiority. Surgical corridor selection should remain individualized according to tumor anatomy, including lateral extension, vascular encasement, and optic canal involvement, as well as institutional expertise and surgeon experience.

**Graphical Abstract:**

**Figure:**
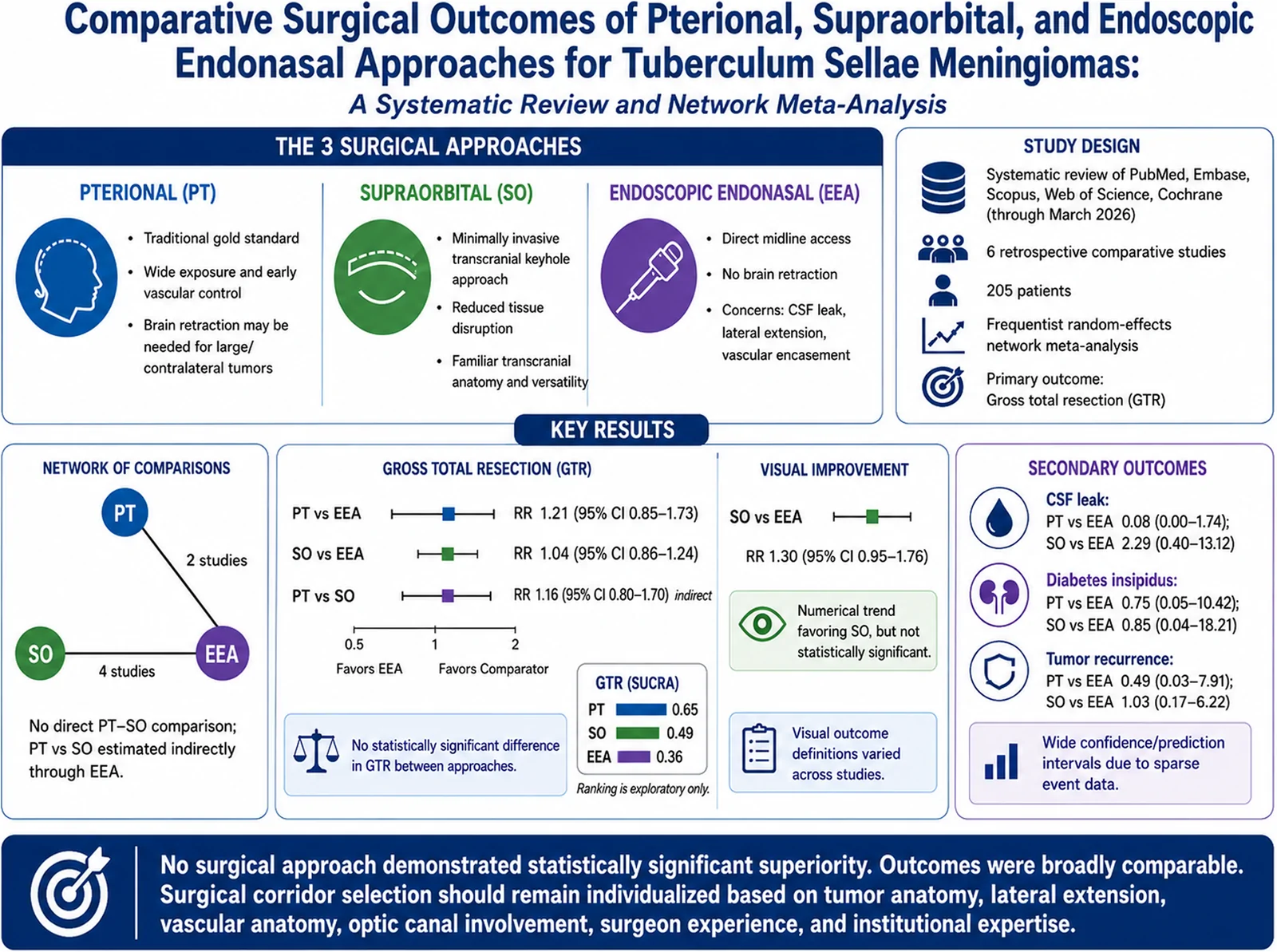

## Highlights

Six retrospective comparative studies involving 205 patients were included.

No surgical approach showed statistically significant superiority for gross total resection.

Visual improvement and complication outcomes were broadly comparable across approaches.

Approach selection should remain individualized according to tumor anatomy and surgical expertise.

## Introduction

Tuberculum sellae meningiomas (TSMs) account for 5–10% of all intracranial meningiomas and typically arise from the dura of the tuberculum sellae, chiasmatic sulcus, and limbus sphenoidale [1]. Their proximity to the optic nerves, optic chiasm, and anterior cerebral circulation makes surgical management uniquely demanding. Timely decompression of the optic apparatus remains critical, as visual recovery is closely related to preoperative function and symptom duration.

The evolution of skull-base surgery has introduced alternative corridors. The endoscopic endonasal transsphenoidal approach offers a direct midline trajectory to the tumor base, enabling inferior optic nerve decompression without brain retraction. Recent comparative studies have reported encouraging visual outcomes with this technique, particularly in small to medium-sized tumors, and meta-analytic data suggest comparable extents of resection in carefully selected patients [2–3]. Nonetheless, concerns regarding CSF leakage, lateral tumor extension, and vascular encasement remain relevant considerations [4, 5].

The central challenge in treating TSMs lies in achieving maximal safe resection while preserving visual and vascular integrity. For decades, transcranial approaches, particularly pterional craniotomy, have served as the mainstay of treatment. These approaches provide a favorable corridor to the optic foramen and optic–carotid recess, facilitating optic nerve decompression, early cerebrospinal fluid drainage, and identification of critical neurovascular structures. However, access to the contralateral side may be limited in large tumors and may require frontal lobe retraction or manipulation [6–8].

In parallel, minimally invasive transcranial techniques such as the supraorbital keyhole approach have sought to combine reduced tissue disruption with the familiarity and versatility of transcranial exposure [7]. Contemporary multicenter analyses emphasize that tumor morphology, optic canal invasion, and vascular involvement are critical determinants of outcome, underscoring that no single approach is universally applicable [8].

Despite expanding surgical options and increasing experience with each technique, the optimal operative strategy for TSMs remains debated. Much of the existing literature consists of retrospective, single-arm series, and direct comparisons across pterional, supraorbital, and endoscopic endonasal approaches within comparable patient populations are limited. As surgical decision-making continues to rely heavily on surgeon preference and institutional practice patterns, systematic comparative evaluation is necessary to better define approach-specific advantages and inform individualized treatment planning.

## Objective

The objective of this study was to comparatively evaluate surgical outcomes of tuberculum sellae meningiomas treated via three approaches: the pterional approach, the endoscopic endonasal transsphenoidal approach, and the supraorbital keyhole approach.

Specifically, we aimed to analyze:

- Gross total resection as the primary outcome
- Postoperative visual outcomes
- Complication profiles, including CSF leak, endocrinopathy, and cranial nerve palsy
- Recurrence rates during follow-up

By systematically comparing these techniques, this study sought to clarify approach-specific advantages and limitations and to provide evidence-based guidance for individualized surgical planning in patients with tuberculum sellae meningiomas.

### Methods

### Search strategy and study selection

A systematic literature search was conducted across major electronic databases using predefined search strings targeting tuberculum sellae meningiomas and surgical approaches (pterional, endoscopic endonasal, and supraorbital).

A comprehensive systematic literature search was conducted in accordance with PRISMA 2020 guidelines to identify comparative studies evaluating surgical approaches for tuberculum sellae meningiomas. The following electronic databases were searched from inception to March 31st, 2026. PubMed/MEDLINE, Embase, Scopus, Web of Science, and the Cochrane Central Register of Controlled Trials (CENTRAL). The search strategy combined controlled vocabulary terms (MeSH in PubMed and Emtree in Embase) with free-text keywords related to tuberculum sellae meningiomas and relevant surgical approaches.

Key controlled vocabulary terms included “Meningioma,” “Skull Base Neoplasms,” “Endoscopy,” “Craniotomy,” and “Transsphenoidal Surgery.” These were combined with free-text terms such as “tuberculum sellae,” “planum sphenoidale meningioma,” “endoscopic endonasal approach,” “EEA,” “pterional approach,” “supraorbital approach,” and “eyebrow keyhole.” Boolean operators (AND/OR), truncation, and field-specific tags were applied to maximize sensitivity. The search strategy was adapted appropriately for each database.

Reference lists of included studies were manually screened to identify additional relevant publications. Citation tracking was performed using Scopus and Web of Science. After removal of duplicates, titles and abstracts were screened independently by two reviewers, followed by full-text assessment of potentially eligible studies. Disagreements were resolved by consensus. Only studies providing extractable comparative data between surgical approaches were included in the final network meta-analysis.

### Eligibility criteria

Studies were included if they evaluated tuberculum sellae meningiomas (TSMs) and compared at least two of the following surgical approaches: pterional (PT), endoscopic endonasal (EEA), or supraorbital (SO). Eligible studies were required to report extractable outcome data at the arm level and provide data on gross total resection (GTR). Non-comparative case series, studies involving mixed skull base cohorts without subgrouped surgical data, and case reports were excluded.

Because outcome definitions varied across the included retrospective studies, outcomes were extracted according to each study’s reported definitions. GTR was generally treated as the authors’ reported complete or gross-total tumor removal, including radiographic or operative definitions when specified. Visual improvement was extracted as reported by each study and could include improvement in visual acuity, visual fields, or composite ophthalmological assessment. This heterogeneity was considered during interpretation and was not assumed to represent fully standardized outcome measurement across studies.

### Risk of bias assessment

Risk of bias was assessed using the Newcastle–Ottawa Scale (NOS) for observational comparative studies. Studies achieving a score of 8-9 were classified a slow risk of bias, whereas those scoring 5-7 were considered to have moderate risk.

Risk-of-bias judgments were incorporated qualitatively into interpretation rather than used for quantitative subgroup analysis because only six studies were eligible. The observational design, retrospective patient selection, incomplete adjustment for anatomical confounders, and variable outcome definitions were considered when grading the certainty of evidence.

### Statistical analysis

A frequentist random-effects network meta-analysis was performed using risk ratios (RRs) as the summary effect measure for dichotomous outcomes. Analyses were conducted in R using the netmeta package. Pooled estimates were calculated using inverse-variance random-effects models. Statistical heterogeneity was assessed using I² and τ² values, and prediction intervals were calculated where applicable. Indirect comparisons were derived under the assumption of transitivity and network consistency. Network geometry was evaluated visually by inspecting available direct comparisons, node size, and edge thickness. Formal statistical testing for inconsistency was limited because the network contained no closed PT–SO–EEA loop and no direct PT versus SO comparison; therefore, inconsistency could not be robustly estimated. Treatment ranking probabilities were estimated using Surface Under the Cumulative Ranking Curve (SUCRA) scores and were interpreted as exploratory only. Sensitivity analyses and meta-regression were not performed because of the small number of included studies, sparse events, and insufficient reporting of key effect modifiers. Publication bias and small-study effects were not formally assessed because fewer than 10 studies were available and each pairwise comparison included too few studies for meaningful funnel-plot or regression-based assessment. The certainty of evidence for the primary outcome was evaluated using the GRADE framework.

## Results

**Fig. 1:**
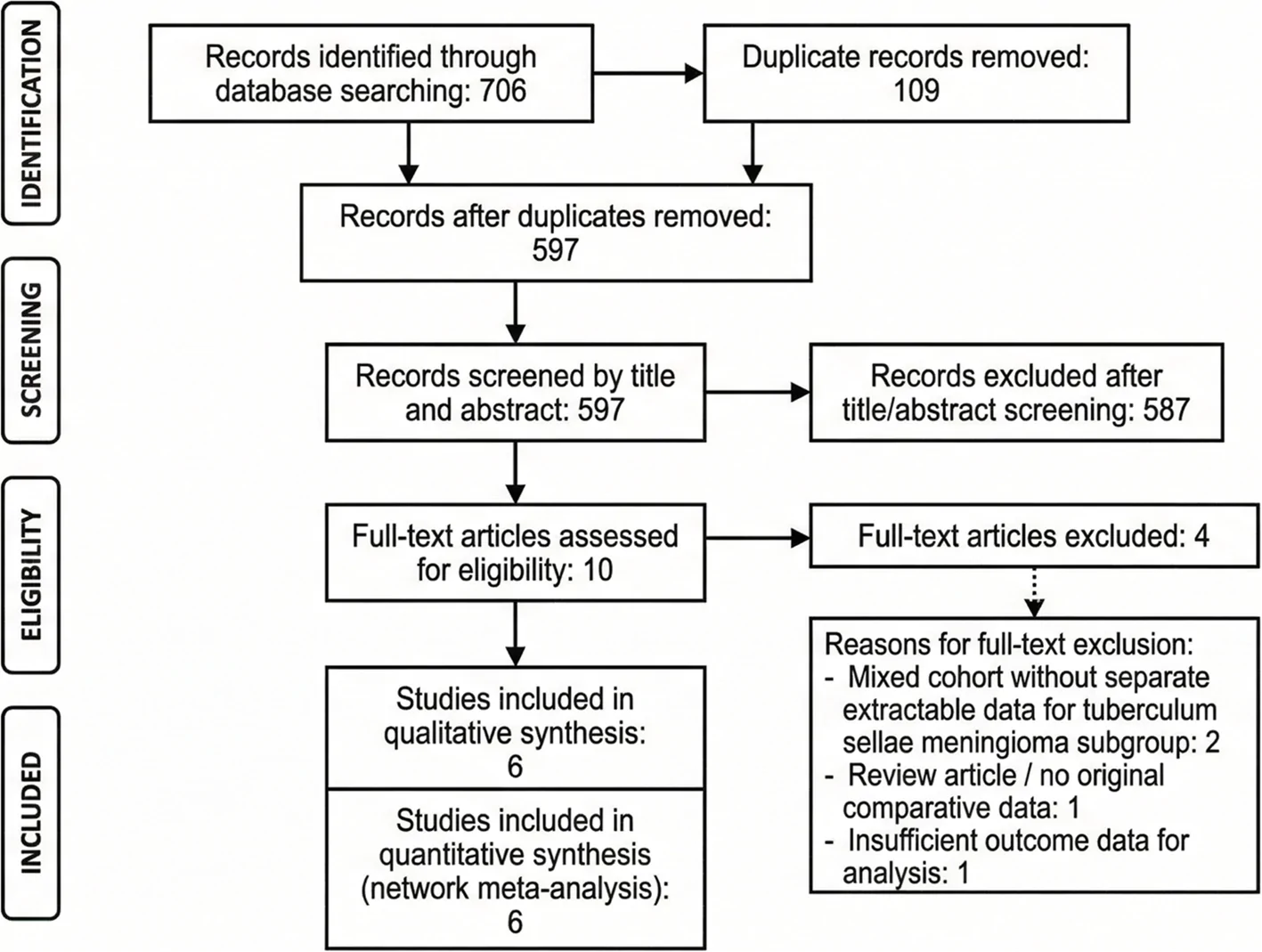
Flowchart summarizing the identification, screening, eligibility assessment, and inclusion of studies evaluating surgical approaches for tuberculum sellae meningiomas

A total of 706 records were identified after application of search strings. After removal of 109 duplicates, 597 studies underwent title and abstract screening. Of these, 587 studies were excluded based on irrelevance, non-comparative design, non-TSM pathology, or lack of surgical subgroup data. The remaining 10 articles underwent full-text review. Four studies were subsequently excluded because cranial surgical approaches were not subgrouped into distinct pterional or supraorbital cohorts, precluding arm-level extraction. Ultimately, 6 retrospective comparative studies met inclusion criteria and were incorporated into the network meta-analysis. A PRISMA flow diagram summarizes the selection process (Fig. 1).

**Fig. 2:**
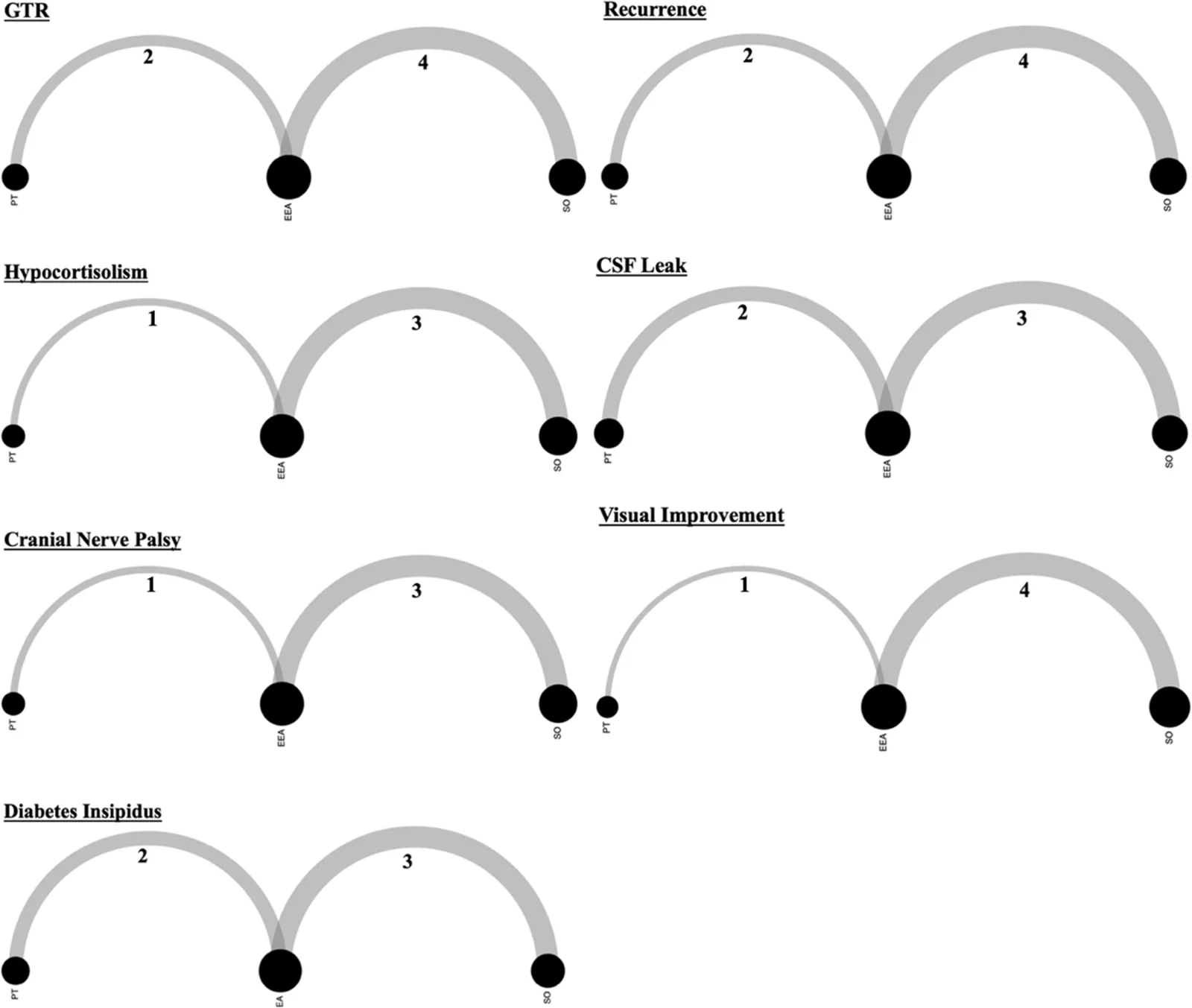
Network geometry of included surgical comparisons. Nodes represent surgical approaches and are scaled according to the number of patients assigned to each approach. Edges represent direct comparative evidence and are scaled according to the number of studies contributing to each comparison. The network included PT–EEA and SO–EEA comparisons; no direct PT–SO comparison was available

Six retrospective comparative studies published between 2011 and 2024 were included, comprising 205 patients with tuberculum sellae meningiomas. All studies compared at least two of the following approaches: pterional (PT), endoscopic endonasal (EEA), and supraorbital (SO) (Fig. 2). Two studies provided PT–EEA comparisons, four provided SO–EEA comparisons, and no direct PT–SO comparisons were available. Consequently, indirect PT–SO comparisons were derived through network meta-analysis with EEA serving as the common comparator (Fig. 2). Among the six included studies, five were rated as moderate risk of bias, while one study (Li et al., 2022) achieved a low-risk rating (Table 1).

**Table 1:** Summary of the six retrospective comparative studies included in the network meta-analysis

Study_ID	Year	Design	Total TSM	PT	EEA	SO	NOS Score (0–9)	Overall Risk
Bowers et al.	2011	Retrospective comparative	27	22	5	NA	6	Moderate
Linsler et al.	2017	Retrospective comparative	22	NA	6	16	7	Moderate
Mallari et al.	2021	Retrospective comparative	33	NA	20	13	6	Moderate
Li et al.	2022	Retrospective comparative	38	NA	7	31	9	Low
Jiang et al.	2023	Retrospective comparative	36	NA	19	17	7	Moderate
Mo et al.	2024	Retrospective comparative	49	23	26	0	6	Moderate

The evidence network was sparse and star-shaped, with EEA serving as the common comparator. No closed loop was present because no study directly compared PT with SO. As a result, the PT–SO estimate was based entirely on indirect evidence, and formal inconsistency assessment was not feasible. These structural limitations were considered when interpreting all network-derived estimates and rankings.

The outcomes were pooled according to the studies in Figs. 3, 4, 5, 6, 7, 8, 9, 10, 11 and 12.

**Fig. 3:**
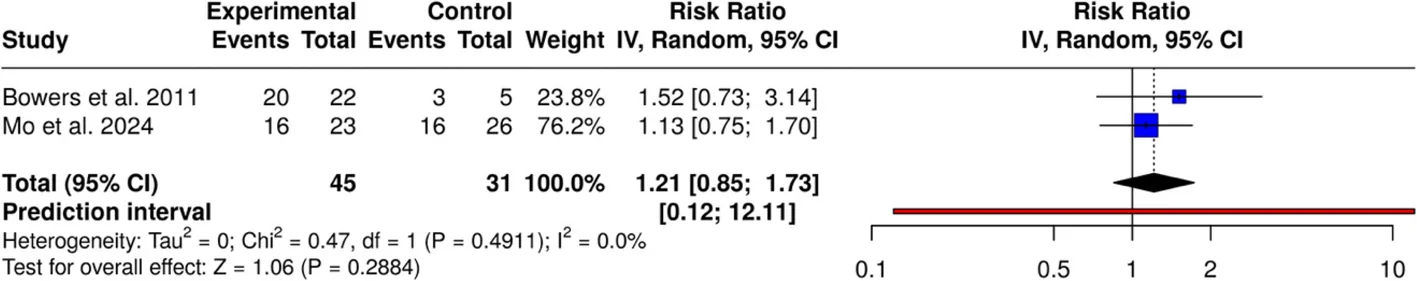
Forest plot of gross total resection (GTR): PT vs EEA

**Fig. 4:**
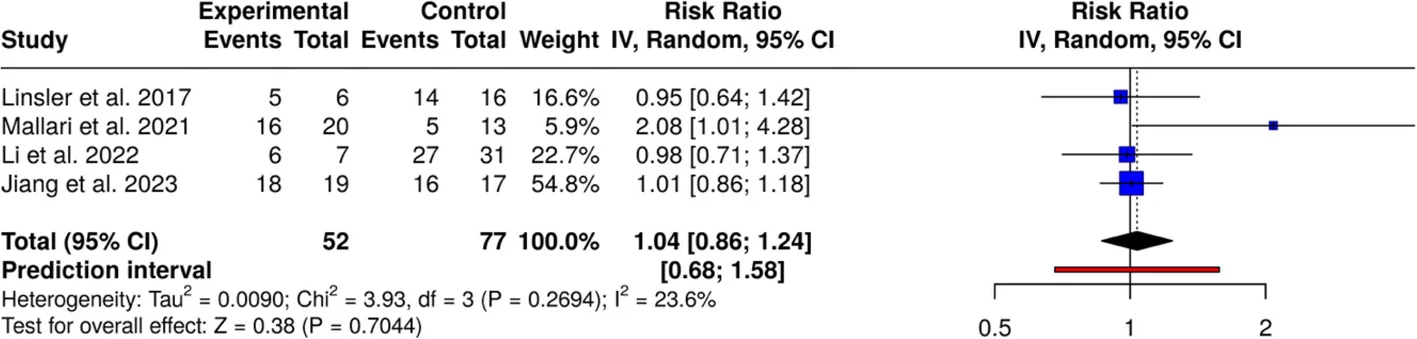
Forest plot of gross total resection (GTR): SO vs EEA

**Fig. 5:**
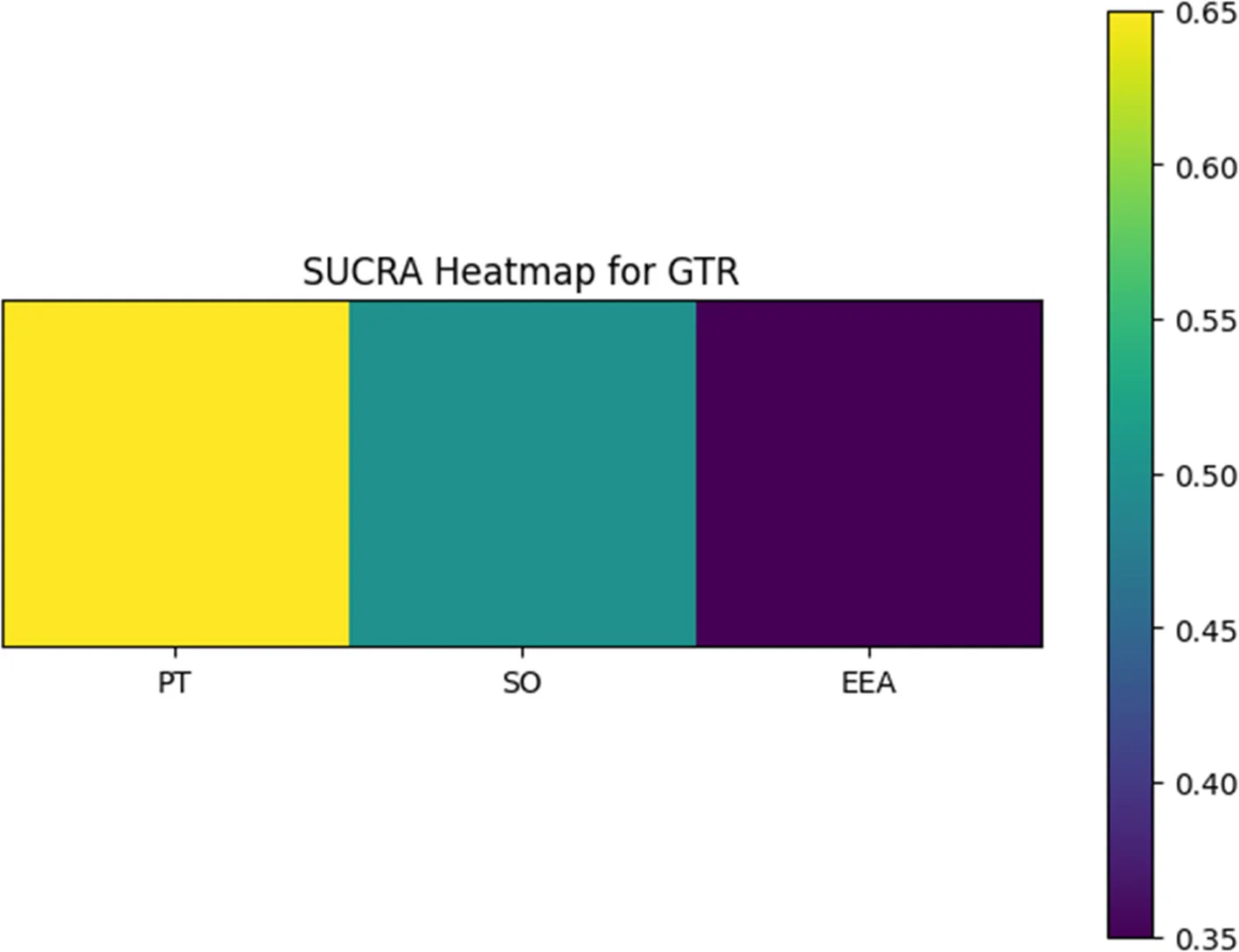
SUCRA ranking plot for gross total resection. Ranking probabilities of the three surgical approaches for achieving gross total resection based on Surface Under the Cumulative Ranking Curve (SUCRA) analysis

**Fig. 6:**
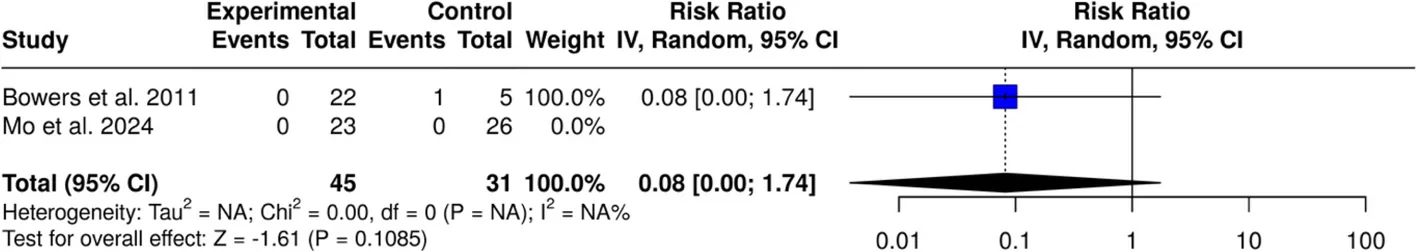
Forest plot showing pooled pairwise comparisons for cerebrospinal fluid leak among PT and EEA

**Fig. 7:**
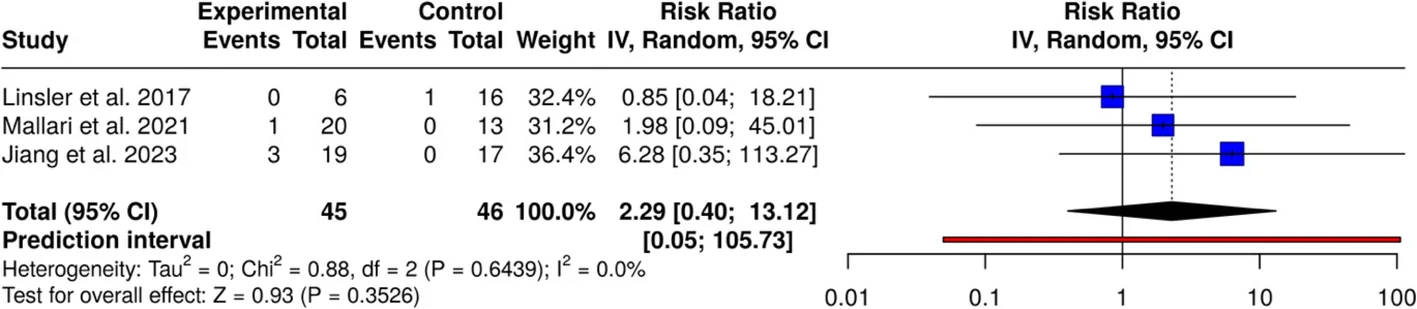
Forest plot showing pooled pairwise comparisons for cerebrospinal fluid leak among SO and EEA

**Fig. 8:**
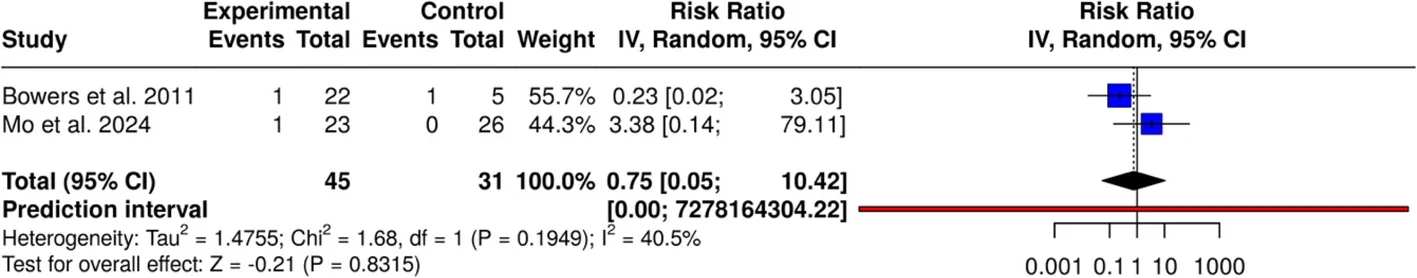
Forest plot showing pooled pairwise comparisons for postoperative diabetes insipidus among PT and EEA

**Fig. 9:**
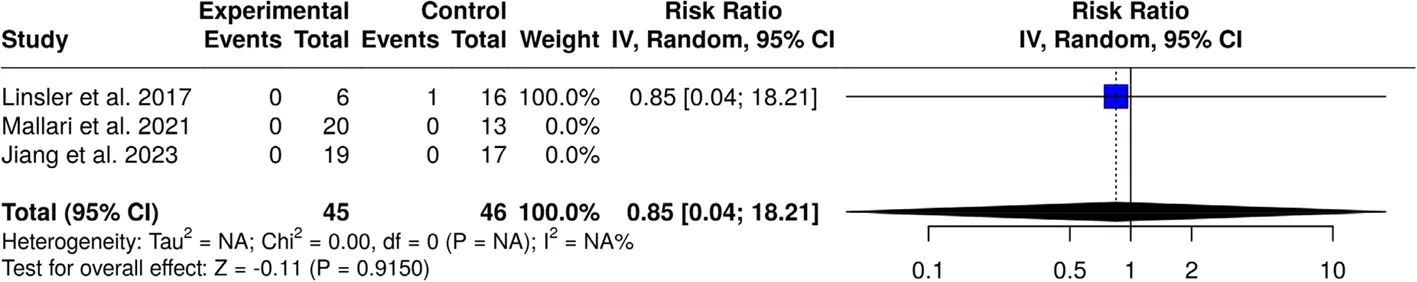
Forest plot showing pooled pairwise comparisons for postoperative diabetes insipidus among SO and EEA

**Fig. 10:**
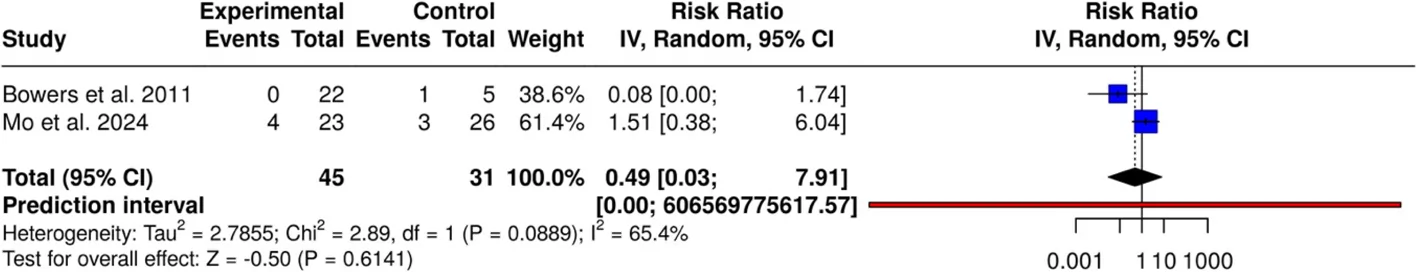
Forest plot showing pooled pairwise comparisons for tumor recurrence among PT and EEA

**Fig. 11:**
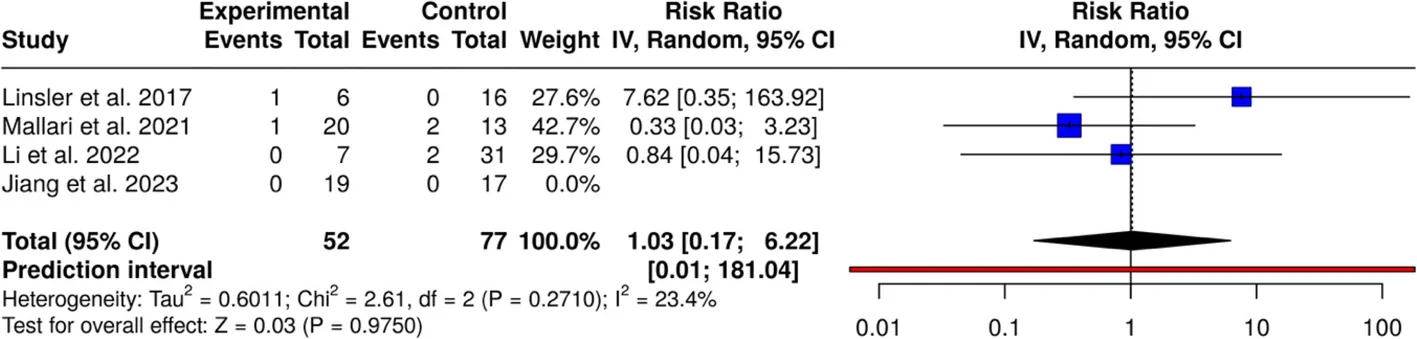
Forest plot showing pooled pairwise comparisons for tumor recurrence

**Fig. 12:**
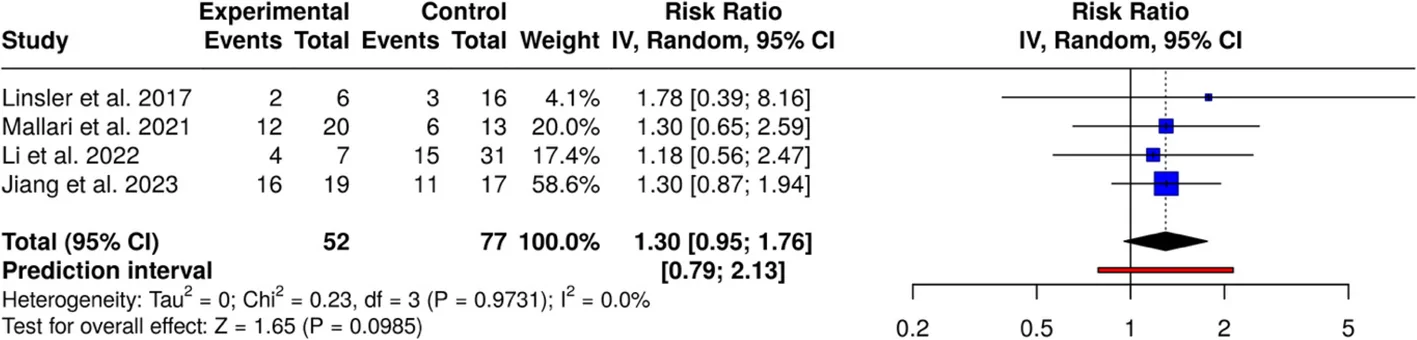
Forest plot of postoperative visual improvement

### Primary outcome: gross total resection (GTR)

#### PT vs. EEA

Two studies were pooled. There was no statistically significant difference in GTR rates between PT and EEA (RR = 1.21, 95% CI 0.85–1.73; *p* = 0.2884). Heterogeneity was negligible (I² = 0%). However, the prediction interval was wide (0.12–12.11), reflecting uncertainty due to limited study numbers.

#### SO vs. EEA

Four studies were included. No statistically significant difference was observed (RR = 1.04, 95% CI 0.86–1.24; *p* = 0.7044). Heterogeneity was low (I² = 23.6%), and the prediction interval ranged from 0.68 to 1.58.

#### Indirect PT vs. SO

The network-derived indirect comparison demonstrated no significant difference between PT and SO (RR = 1.16, 95% CI 0.80–1.70).

### Treatment ranking

SUCRA analysis suggested an exploratory numerical ranking in which PT had the highest probability of ranking first for GTR, followed by SO and EEA. The SUCRA heatmap showed only modest separation between PT, SO, and EEA. However, because all pairwise comparisons were non-significant, confidence intervals overlapped, and the PT–SO comparison was indirect, these rankings should not be interpreted as evidence of clinical superiority. Overall, the ranking results are hypothesis-generating and do not establish that any surgical approach achieves higher GTR rates.

### Secondary outcomes

#### Cerebrospinal fluid (CSF) leak

##### PT vs. EEA

Two studies were pooled. Although the point estimate favored PT (RR = 0.08), the result did not reach statistical significance (95% CI 0.00–1.74; *p* = 0.1085). The extremely wide confidence interval reflects sparse event data.

##### SO vs. EEA

Three studies were included. No significant difference was observed (RR = 2.29, 95% CI 0.40–13.12; *p* = 0.3526), with no detected heterogeneity (I² = 0%). The prediction interval was notably wide (0.05–105.73), indicating substantial imprecision.

#### Diabetes insipidus (DI)

##### PT vs. EEA

Two studies were included. No statistically significant difference was observed (RR = 0.75, 95% CI 0.05–10.42; *p* = 0.8315). The prediction interval was extremely broad, reflecting low event counts.

##### SO vs. EEA

Three studies contributed data. No significant difference was detected (RR = 0.85, 95% CI 0.04–18.21; *p* = 0.9150). Heterogeneity could not be reliably estimated due to sparse data.

#### Tumor recurrence

##### PT vs. EEA

Two studies were pooled. No significant difference in recurrence was observed (RR = 0.49, 95% CI 0.03–7.91; *p* = 0.6141). Moderate heterogeneity was present (I² = 65.4%), and the prediction interval was extremely wide.

##### SO vs. EEA

Three studies were included. The pooled estimate showed no statistically significant difference (RR = 1.03, 95% CI 0.17–6.22; *p* = 0.9750), with low heterogeneity (I² = 23.4%).

#### Visual Improvement

Four studies contributed to the SO vs. EEA comparison. No statistically significant difference was observed (RR = 1.30, 95% CI 0.95–1.76; *p* = 0.0985), with no heterogeneity (I² = 0%). The prediction interval ranged from 0.79 to 2.13. Although the point estimate favored SO, statistical significance was not reached.

Definitions of visual improvement varied across studies and were not uniformly based on the same ophthalmological measures or timing of postoperative assessment. Some studies emphasized visual acuity, others visual fields or combined visual status. Therefore, the pooled visual outcome should be interpreted as a study-defined composite rather than a fully standardized endpoint.

#### Hypocortisolism and cranial nerve palsy

Sparse event data were available. The pooled comparison between SO and EEA did not demonstrate a statistically significant difference. Estimates were imprecise with wide confidence intervals, reflecting low event frequency.

## Discussion

This systematic review and network meta-analysis compared pterional, supraorbital keyhole, and endoscopic endonasal approaches for tuberculum sellae meningiomas within a unified analytical framework. The available evidence was limited to six retrospective comparative studies comprising 205 patients. No approach demonstrated statistically significant superiority for GTR, visual improvement, recurrence, or complications. Accordingly, the results should be interpreted as exploratory and hypothesis-generating rather than definitive comparative evidence. Numerical trends and SUCRA rankings may help identify patterns for future research but should not be used alone to infer superiority of one approach over another.

### Gross total resection

Achieving GTR remains the primary surgical goal in TSM management, as it is closely associated with reduced recurrence risk and improved long-term outcomes. The numerical SUCRA pattern favoring PT for GTR may reflect anatomical and selection factors rather than a true treatment effect. The PT corridor provides broad lateral exposure and may be better suited for tumors with marked lateral extension or vascular involvement. However, the absence of statistically significant pairwise differences, the lack of direct PT–SO evidence, and the retrospective nature of all included studies preclude any conclusion that PT is superior for GTR.

The trend favoring transcranial approaches for GTR can be explained by anatomical considerations. The PT corridor provides broad lateral exposure and allows early identification and mobilization of critical neurovascular structures, including the optic nerve and the internal carotid artery. This panoramic visualization facilitates resection of tumors with substantial lateral extension beyond the optic nerves and vascular encasement, features that are commonly regarded as important limitations or relative contraindications for the endonasal route. Optic canal involvement alone should not be considered a contraindication to EEA, because inferomedial optic canal decompression is one of the potential advantages of the endonasal corridor in appropriately selected tumors [9, 10]. The SO approach, which is a minimally invasive keyhole approach, shares many of these advantages while offering reduced soft tissue disruption, and prior single-institution series have reported GTR rates comparable to standard pterional craniotomy in appropriately selected patients [11]. The reduced working corridor associated with a minicraniotomy may limit surgical maneuverability and may lead to a lower SUCRA score compared with PT, although the use of endoscopic assistance with angled optics can help overcome this constraint [12].

In contrast, the EEA provides a direct midline corridor to the tumor base with favorable access to the inferomedial optic nerve and the interoptic space. While this can facilitate early devascularization and decompression of the optic apparatus, EEA is inherently constrained by its inability to address tumors extending significantly lateral to the optic nerves or encasing major vascular structures. The lower SUCRA ranking for GTR via EEA in our analysis aligns with this anatomical limitation. However, these ranking probabilities should be interpreted with caution given the overlapping confidence intervals and absence of statistical significance across all pairwise comparisons. This is consistent with prior meta-analyses, which generally reported comparable GTR rates between endonasal and transcranial approaches (Agosti et al. 2024 [4]; Lee et al. 2026 [5]), suggesting that no approach demonstrates clear superiority in achieving GTR when studied in pooled comparative analyses.

Nevertheless, a critical methodological caveat must be considered when interpreting GTR comparisons across approaches. There remains a consistent clinical tendency to reserve transcranial approaches for anatomically complex tumors, those with large dimensions, lateral extension, vascular encasement, or optic canal invasion, precisely the cases where GTR is most difficult to achieve [7]. This inherent selection bias renders direct comparisons potentially unfair. Magill et al. [8] demonstrated that lower optic canal invasion score and transcranial approach were both independently associated with GTR on multivariate analysis, confirming that even after accounting for tumor complexity, the transcranial approach retains an independent association with higher rates of GTR. The authors accordingly emphasized that approach selection should be individualized based on tumor anatomy, surgeon experience, and patient expectations.

Importantly, the absence of statistical significance across all pairwise comparisons likely reflects the limited number of included studies and the heterogeneity of tumor characteristics, patient selection, and institutional expertise across contributing centers, all of which are important confounders in this literature.

### Visual outcomes

Visual impairment is the most common presenting symptom of TSMs, and postoperative visual improvement is often the primary outcome of interest for patients and clinicians alike [14]. In our analysis, the SO vs. EEA comparison for visual improvement demonstrated a numerical trend favoring SO (RR = 1.30, 95% CI 0.95–1.76; *p* = 0.0985), though this did not reach statistical significance, with zero heterogeneity across four contributing studies and a prediction interval of 0.79 to 2.13. There was no direct comparison available between PT and EEA, or between the two transcranial approaches, representing an important gap in the current evidence base.

This finding stands in apparent contrast to the broader literature, where EEA has been advocated by several groups as offering superior visual outcomes owing to several inherent anatomical and technical advantages [7, 14]. Approaching the tumor from below provides a direct trajectory to the optic canal, enabling up to 270° early circumferential decompression, particularly beneficial in cases of optic canal invasion, where complete intracanalicular tumor removal may be achieved while simultaneously reducing the risk of recurrence [14]. Additionally, the close-up, magnified, and high-definition endoscopic view facilitates bimanual microdissection with direct visualization of the surgical plane between the tumor and the optic chiasm and vascular perforators, potentially minimizing the blind manipulation that may contribute to postoperative visual deterioration via transcranial routes. These advantages have been highlighted in recent comparative studies and meta-analyses reporting favorable visual improvement rates with EEA, particularly for small- to medium-sized tumors [5, 11, 13].

However, several factors may explain the discrepancy between these findings and our results.

First, most prior studies comparing visual outcomes have evaluated EEA against all transcranial approaches collectively, without distinguishing between different techniques. The supraorbital keyhole approach, as a distinct minimally invasive transcranial corridor, may offer visual outcome advantages over the standard TC approaches by virtue of its more limited brain exposure and reduced optic nerve manipulation, therefore grouping it with the TC approaches in prior analyses may have underestimated the visual outcomes.

Second, significant technical heterogeneity exists within TC approaches themselves, particularly regarding optic canal management. The decision to perform optic canal unroofing, and critically, its timing relative to tumor removal, whether performed before, during, or after resection, varies considerably across surgeons and institutions and has been shown to influence visual outcomes [15–18]. The timing of optic nerve decompression appears to be a particularly important determinant of visual outcome. The rationale for early optic canal decompression, performed before tumor resection begins, lies in releasing tension on the compressed nerve prior to any surgical manipulation, thereby minimizing the risk of additional ischemic or mechanical injury during tumor dissection. This strategy has been associated with superior visual improvement rates and reduced intraoperative visual deterioration, as demonstrated by Nozaki et a. [15]. and Otani et al. [13], whose results appearedmore favorable than series inwhich optic canal explorationwas performed at the end ofresection or not at all [16–21]. This variability is rarely captured or adjusted for in comparative studies, introducing an important source of heterogeneity that may confound cross-approach visual outcome comparisons.

Third, as discussed in the context of GTR, there remains a consistent tendency to select EEA for smaller, less complex tumors and transcranial approaches for larger tumors with lateral extension or vascular encasement. Since preoperative visual impairment severity and tumor size are among the strongest predictors of visual recovery [22], this selection bias might influence visual outcome comparisons in a direction that may artificially favor EEA in the broader literature.

An additional limitation is the lack of standardized visual outcome definitions across included studies. Visual acuity, visual field recovery, composite visual improvement, and timing of postoperative assessment were not consistently reported. These differences may introduce outcome misclassification and reduce confidence in the pooled visual improvement estimate.

In summary, while EEA offers compelling anatomical and technical advantages for optic nerve decompression, the visual outcome comparison across approaches remains confounded by several important factors, including the lack of distinction between transcranial techniques in prior literature, significant institutional and surgeon variability in optic canal management and its timing, and the persistent tendency to select EEA for less complex tumors. These confounders collectively limit the validity of cross-approach visual outcome comparisons. Taken together, future prospective studies incorporating propensity-score matching or a validated TSM grading scale will be essential.

#### Recurrence

Tumor recurrence following TSM resection is primarily driven by the extent of resection, with Simpson grade being the most established predictor of long-term tumor control. In the broader literature, recurrence rates following surgical resection of TSMs have generally been reported as low, though follow-up durations vary considerably [8]. Importantly, the observed recurrence rate was 10% in the Magill et al. series, with no significant difference between transcranial and transsphenoidal approaches, a finding consistent with the larger International TSM Study Group analysis, which similarly demonstrated no difference in the overall recurrence rates between EEA and TCA [23]. In our analysis, no statistically significant difference in recurrence rates was observed between any of the compared approaches, neither PT vs. EEA (RR = 0.49, 95% CI 0.03–7.91) nor SO vs. EEA (RR = 1.03, 95% CI 0.17–6.22), consistent with this pattern. However, these results must be interpreted with considerable caution, as event counts were extremely sparse, heterogeneity in the PT vs. EEA comparison was moderate (I² = 65.4%), and follow-up duration varied considerably across included studies. Given that recurrence in WHO grade I meningiomas may manifest years to decades after initial resection, the short to medium-term follow-up available in the current evidence base is insufficient to draw definitive conclusions about long-term tumor control across approaches.

### Complications

One of the most clinically meaningful aspects of approach selection in TSM surgery concerns the complication profile, as each corridor carries distinct anatomical risks. Our analysis found no statistically significant differences between approaches for any of the assessed complications, including CSF leak, diabetes insipidus, hypocortisolism, and cranial nerve palsy. However, the estimates for these outcomes were imprecise, characterized by wide confidence intervals and very low event counts, limiting any meaningful interpretation.

Cerebrospinal fluid leak represents one of the most frequently cited concerns specific to the EEA [7] and has historically been considered a significant limitation of the endonasal corridor. Early meta-analyses reported CSF leak rates as high as 32% [24] and 21% [25] following EEA for TSMs. However, the evolution of skull base reconstruction techniques, particularly the introduction of the vascularized Hadad-Bassagasteguy nasoseptal flap, gasket seal closure, multilayer reconstruction, and judicious use of lumbar drainage, has significantly reduced this risk, with reported rates declining from approximately 22% between 2004 and 2010 to as low as 4% between 2016 and 2020 [26]. This significant improvement in reconstructive technique has broadened the applicability of EEA in appropriately selected TSM patients. In our analysis, although the point estimate for CSF leak favored PT over EEA (RR = 0.08), this did not reach statistical significance (95% CI 0.00–1.74; *p* = 0.11), and no significant difference was observed between SO and EEA (RR = 2.29, 95% CI 0.40–13.12). The absence of statistical significance across both comparisons likely reflects the sparse event data within the included studies rather than true equivalence, and the wide confidence intervals preclude any definitive conclusions regarding approach-specific CSF leak risk.

Endocrine complications, including diabetes insipidus and hypocortisolism, are generally infrequent following surgery for TSMs regardless of the approach employed [17, 26]. Although the endonasal corridor theoretically carries a higher risk of pituitary dysfunction due to its closer anatomical proximity to the pituitary gland, stalk, and superior hypophyseal vasculature, neither our analysis nor the broader comparative literature have substantiated this theoretical concern. In our analysis, no statistically significant differences were observed between surgical approaches with respect to these complications, with all comparisons yielding wide confidence intervals reflecting very low event counts. This finding is consistent with several comparative studies and pooled analyses, which have generally failed to demonstrate clear superiority of one approach over another in terms of pituitary dysfunction [14, 23].

Operative duration was not suitable for pooled analysis in the present review because it was inconsistently reported across the included comparative studies. Nevertheless, operative time is clinically relevant when comparing TSM approaches, particularly because longer procedures may influence perioperative morbidity, resource utilization, and surgeon preference. Future comparative studies should report operative duration using standardized definitions and should adjust for tumor size, extension, reconstruction requirements, and surgeon experience.

In summary, our findings suggest that no single surgical approach demonstrates clear superiority in the management of tuberculum sellae meningiomas across the evaluated outcomes. While SUCRA-based ranking indicated a potential advantage of transcranial approaches for gross total resection, these findings were not supported by statistically significant differences in pairwise comparisons. These results underscore the need for a tailored, anatomy-driven approach to surgical decision-making.

### Limitations

Several important limitations must be acknowledged. First, the analysis is based exclusively on retrospective comparative studies, most of which had moderate risk of bias, and the total sample size was modest at 205 patients across six studies. Second, several outcome analyses were limited to two or three studies with sparse event counts, producing wide confidence and prediction intervals and limiting statistical power. Third, no direct PT versus SO comparison was available, leaving PT–SO estimates dependent on indirect evidence through EEA. Because the network contained no closed loop, formal inconsistency testing could not be performed. Fourth, key confounders, including tumor size, lateral extension, optic canal involvement, vascular encasement, tumor consistency, preoperative visual status, and surgeon experience, were not uniformly reported or adjustable across studies. Fifth, outcome definitions were heterogeneous, particularly for GTR and visual improvement, reducing certainty in pooled estimates. Sixth, sensitivity analyses, subgroup analyses, meta-regression, and publication-bias assessment were not feasible because of the small number of studies and sparse events. Finally, institutional variation in surgical expertise, particularly for EEA and skull-base reconstruction, may have influenced outcomes. These limitations support cautious interpretation of all pooled estimates and SUCRA rankings.

## Conclusions

This network meta-analysis found no statistically significant differences among pterional, supraorbital, and endoscopic endonasal approaches for tuberculum sellae meningiomas across GTR, visual improvement, recurrence, or complication outcomes. Numerical SUCRA rankings suggested possible trends, but these findings are exploratory and should be interpreted cautiously because of the small retrospective evidence base, sparse events, heterogeneous outcome definitions, and absence of direct PT–SO comparisons. Current evidence supports individualized, anatomy-driven approach selection based on tumor morphology, lateral extension, vascular anatomy, optic canal involvement, surgeon experience, and institutional expertise. Larger prospective comparative studies with standardized outcome definitions, operative-duration reporting, and patient-level adjustment for anatomical confounders are needed.

## Data Availability

This is a network meta-analysis based on previously published data.

## Abbreviations

TSM: Tuberculum sellae meningioma
PT: Pterional approach
SO: Supraorbital approach
EEA: Endoscopic endonasal approach
GTR: Gross total resection
CSF: Cerebrospinal fluid
DI: Diabetes insipidus
RR: Risk ratio
NOS: Newcastle–Ottawa Scale
SUCRA: Surface under the cumulative ranking curve
